# Photodynamic therapy in combination with immune checkpoint inhibitors plus chemotherapy for first-line treatment in advanced or metastatic gastric or gastroesophageal junction cancer: A phase 2–3 clinical trial protocol

**DOI:** 10.3389/fphar.2023.1063775

**Published:** 2023-01-26

**Authors:** Yang Yu, Rong Yu, Na Wang, Yuping Bai, Qianling Shi, Ewetse Paul Maswikiti, Hao Chen

**Affiliations:** ^1^ The Department of Tumor Surgery, Lanzhou University Second Hospital, Lanzhou, China; ^2^ The Second Clinical Medical College, Lanzhou University, Lanzhou, China; ^3^ The First Clinical Medical College, Lanzhou University, Lanzhou, China

**Keywords:** photodynamic therapy, immune checkpoint inhibitor, chemotherapy, clinical trials, gastric cancer

## Abstract

**Introduction:** The immune checkpoint inhibitor (ICI) has been approved as the first-line therapy for metastatic gastric cancer in China. The treatment response of immune checkpoint inhibitor is highly dependent on the immune condition within the tumor microenvironment. Photodynamic therapy (PDT) has a long history in cancer treatment, and recent studies showed it had an immunomodulatory effect on the tumor. Here we will conduct a trial to assess whether or not a combination with Photodynamic therapy will improve the outcomes of immune checkpoint inhibitor-based treatment in patients with advanced or metastatic gastric cancer.

**Methods:** This study is a single-center, open-label, randomized controlled, phase 2–3 trial. Patients (18–65 years old) with untreated gastric or gastroesophageal junction adenocarcinoma will be eligible for this trial. Sixty participants will be enrolled and randomly divided into the test group (*n* = 30) and control group (*n* = 30) to receive photodynamic therapy in combination with immune checkpoint inhibitor plus chemotherapy and immune checkpoint inhibitor plus chemotherapy, respectively. The primary is progression-free survival (PFS). The secondary outcomes include objective response rates (ORRs) and the occurrence of adverse events. In addition, we will also assess the changes in peripheral blood mononuclear cells (PBMCs) and tumor microenvironment after photodynamic therapy treatment in the test group. Evaluation of the tumor response will be performed every two cycles for a maximum of eight cycles.

**Discussion:** Photodynamic therapy has an immunomodulatory effect on the tumor microenvironment; however, this has not been demonstrated for gastric cancer in a clinical trial. Based on our experience of photodynamic therapy treatment in digestive tract tumors, we plan to conduct a randomized controlled trial on this topic. This will be the first study to evaluate the synergistic effect of photodynamic therapy with immunochemotherapy for patients with advanced gastric cancer.

**Ethics and dissemination:** It was approved by the Institutional Research Ethics Committee of Lanzhou University Second Hospital (No. 2022A-491). When this trial is completed, it will be shared at conferences and submitted for a potential publication in a peer-reviewed journal.

**Clinical Trial Registration:** http://www.chictr.org.cn/, identifier ChiCTR2200064280.

## 1 Introduction

Gastric cancer, including gastroesophageal junction cancer, is one of the most common digestive tract malignancies. According to the 2020 Global Cancer Statistics report, there were 1,089,103 new cases of gastric cancer and 768,793 deaths worldwide in 2020 (Sung et al., 2021). As its dormant symptoms in an early phase, most patients have already suffered an advanced disease when they are diagnosed (Wagner et al., 2017). Patients with advanced or metastatic gastric cancer have a poor prognosis with a 5-year survival rate below 20% (Yang et al., 2020; Kahraman and Yalcin, 2021).

Immunotherapy with immune checkpoint inhibitors (ICIs) has achieved great success in cancer management due to its wide range of applications and good safety. In China, ICI plus chemotherapy has currently been recommended as first-line treatment for metastatic gastric cancer (Committee, 2022). However, the efficacy of ICIs is significantly dependent on the immune microenvironment with tumor tissues in each patient. In gastric cancer, patients with a high PD-L1 expression (CPS ≥ 5) could have superior benefits than those with low PD-L1 (CPS ≥ 1) (Janjigian et al., 2021). PD-L1 is thus used to select patients for ICIs treatment in clinical practice because it can reflect the immune microenvironment to some extent. However, most patients with gastric cancer have a poor immune condition that cannot meet the criterion of CPS ≥ 5 for ICIs usage. Thus, it will be of significance to find a measure to improve a patient’s tumor microenvironment and enhance the effects of ICIs.

Photodynamic therapy (PDT) is a local therapy for tumors, which has a history of almost 30 years (Dougherty, 1993). PDT uses a photosensitizer activated by light to kill cancer cells, which is achieved by the production of oxygen radicals. For gastric cancer, PDT is mainly used for early disease. It is not commonly used in advanced gastric cancer as its limited depth of irradiation. In recent years, an immunomodulatory effect of PDT was found in cancer conditions. PDT can induce a systematic immune response and lead to immunogenic cell death of the tumor cells (Beltrán Hernández et al., 2020). With the development of material science, a lot of novel drug delivery systems have been designed to achieve multifunctional combined therapies (Cheng et al., 2020; Fan et al., 2021; Chen et al., 2022; Cheng et al., 2022). Very recently, some studies have evaluated the feasibility of photoimmunotherapy by encapsulating photosensitizers and immunostimulatory drugs into one micelle and revealed a synergistic effect of these combined strategies in inducing the immunoresponsive tumor microenvironment (Chen et al., 2022; Cheng et al., 2022). This means PDT has the potential to help recruit immune cells into the tumor microenvironment and improve the effects of immunotherapy.

Here, we plan to perform a phase 2–3 open-label clinical trial by studying the outcomes of PDT in combination with ICI plus chemotherapy versus ICI plus chemotherapy, aimed to determine the clinical value of PDT as an adjuvant for improving ICIs-based treatment in advanced or metastatic gastric cancer and gastroesophageal junction cancer.

## 2 Methods and analysis

### 2.1 Study design

**FIGURE 1:**
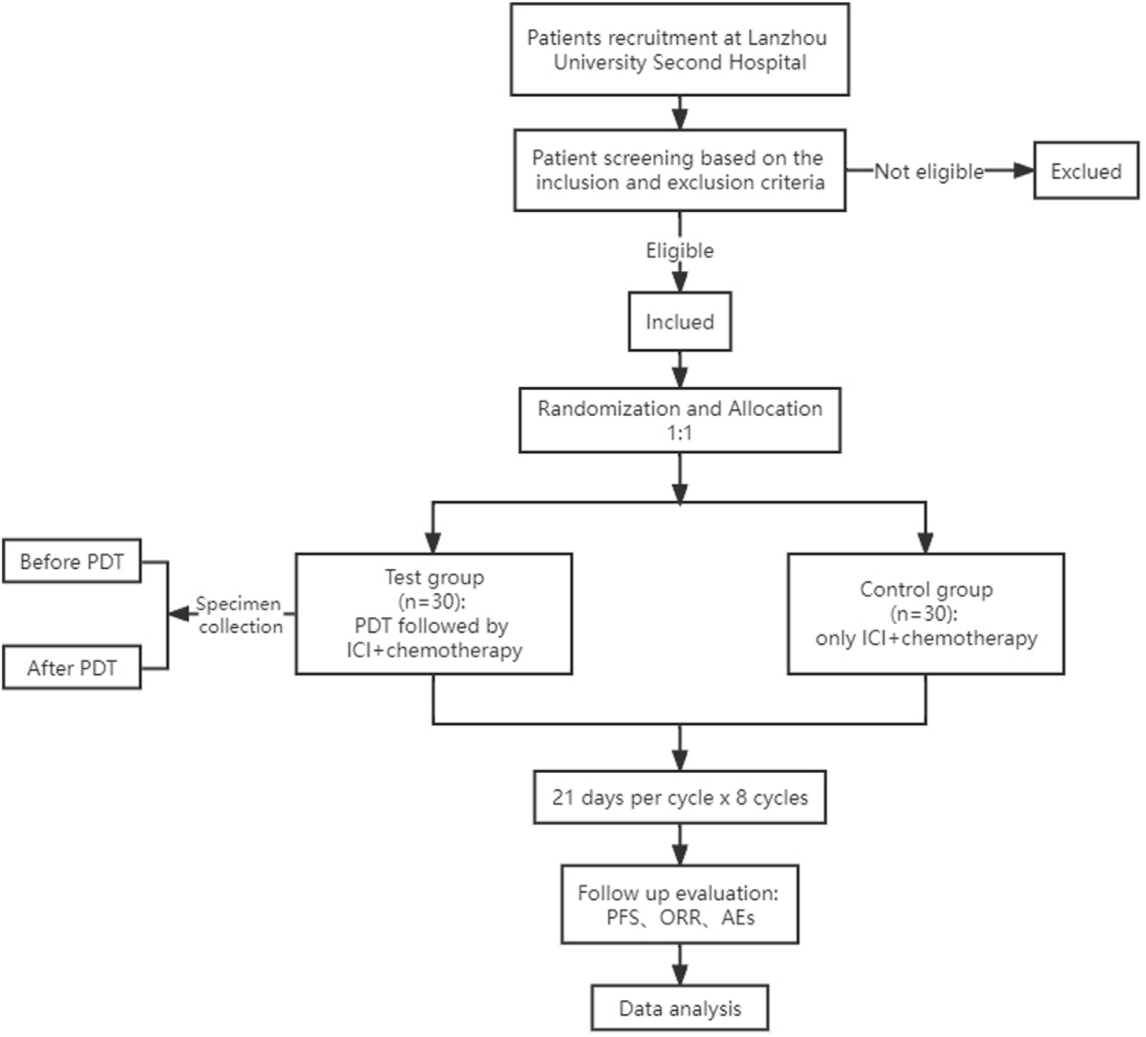
Flowchart of the trial. PDT, photodynamic therapy; ICI, immune checkpoint inhibitor; PFS, progression-free survival; ORR, objective response rate; AEs, adverse events.

This study is a single-center, open-label, 1:1 randomized, phase 2–3 clinical study in which we will evaluate the efficacy and safety of PDT in combination with immunochemotherapy for untreated gastric cancer (Figure 1). Meanwhile, we will also collect the specimens of tumor tissue and blood before and after PDT treatment to explore the PDT-induced changes in the immune system and the tumor microenvironment in patients with gastric cancer.

### 2.2 Study setting

The study will be conducted at the Cancer Center of Lanzhou University Second Hospital. It is expected to complete in 18 months once initiated.

### 2.3 Inclusion criteria

All patients who meet the following criteria will be enrolled: 1) 18–65 years old; 2) untreated gastric or gastroesophageal junction adenocarcinoma confirmed by histopathological examination; 3) advanced or metastatic stage; 4) ECOG score 0–2; 5) expected survival >6 months; 6) having at least one measurable lesion; 7) adequate bone marrow, kidney and liver function defined as neutrophil count ≥1.5 × 10^9^/L, platelet count ≥100 × 10^9^/L, creatinine ≤1.5 times the upper limit of normal (ULN), total bilirubin ≤1.5 times the ULN, and aspartate transaminase and alanine aminotransferase ≤5.0 times the ULN; 8) and providing signed informed consent.

### 2.4 Exclusion criteria

Any participants will be excluded when they have one of the following conditions: 1) multiple primary tumors; 2) prior usage of immunomodulator drugs or any medicines having a direct impact on the immune system; 3) pregnancy or lactation for women; 4) history of allergy to the tested medications or light sensitiveness; 5) contraindication to gastroscopy; 6) comorbidities including autoimmune disease, severe cardiovascular disease, mental illness or severe neurosis, gastrointestinal bleeding and other severe diseases; 7) other inappropriate condition for inclusion determined by the researchers.

### 2.5 Study interventions

Included patients will be divided into the test group and control group. In the test group, patients will receive a PDT operation followed by ICI plus chemotherapy. In the control group, only ICI plus chemotherapy will be performed. 48 h before the PDT procedure, a photosensitizer (hematoporphyrin injection, Hiporfin^®^) will be intravenously injected into the patients in the test group. The dose of photosensitizer is determined based on the weight of patients after carefully evaluating the patient’s kidney and liver function (3–5 mg/kg). Patients with injected photosensitizer will be kept in the dark ward to avoid light exposure until the PDT operation. During the PDT operation, basal anesthesia will be given to the patient first. Then, a gastroscope-based PDT treatment will be administered with a 630 nm laser light delivered by a cylindrical fiber. The cylindrical fiber will be placed near the tumor to ensure complete illumination, and a multi-fractions illumination may be used in some cases (e.g., the tumor is too large to illuminate within one fraction of illumination). The laser light will be generated by the semiconductor laser photodynamic therapy instrument (PDT 630II, Guilin Xingda Photoelectric Medical Equipment Co., Ltd). The parameter of illumination will be set as 800 mW for 15 min with a total energy of 720J. Considering the advanced patients we will include, the patients will receive PDT illumination for four continuous days with the same procedure. After the PDT treatment, patients will be avoided direct light exposure for at least 1 month. In the second week after PDT, patients will receive ICI (Sintilimab or Camrelizumab, 200 mg, day 1, every 3 weeks) plus chemotherapy [SOX (Tegafur gimeracil oteracil potassium capsule twice a day, day 1–14, and oxaliplatin 130 mg/m^2^, day 1, every 3 weeks) or XELOX (capecitabine 1,000 mg/m^2^ twice a day, days 1–14, and oxaliplatin 130 mg/m^2^, day 1, every 3 weeks)]. Tegafur gimeracil oteracil potassium capsule and capecitabine will be orally administered, and others will be intravenously administered. PDT will be only used in the first cycle of treatment for the patients in the test group, which means only ICI plus chemotherapy will continue in the following cycles of treatment. For patients in the control group, the same ICI plus chemotherapy scheme will be given, but no prior PDT operation will be performed in the first treatment cycle.

### 2.6 Termination of the trial

All regimens will continue until confirmed disease progression, unacceptable toxicity, drop out, or the study end. The study end for a subject is defined as treatment up to eight cycles. After eight cycles, patients will continue the treatment or change a new scheme according to the judgment of the investigator.

### 2.7 Outcomes

The primary outcomes are objective response rate (ORR) and progression-free survival (PFS). The best overall response from ICI plus chemotherapy initiation to disease progression or last follow-up will be used to analyze the ORR. ORR refers to the proportion of patients with complete response (CR) and partial response (PR). PFS is defined as the time from ICI plus chemotherapy initiation to the date of first documented tumor progression or death no matter which happens first. All evaluations of treatment response or progression will be conducted by experienced radiologists according to the RECIST 1.1 criteria (Eisenhauer et al., 1990). The safety will be assessed using the incidence of adverse events defined as any toxicity from intervention initiation to the study end according to the NCI-CTCAE 5.0 criteria (Institute, 2017). For patients who receive PDT treatment, a self-control assessment before and after PDT will be performed, in which the changes in peripheral blood mononuclear cells (PBMCs) and intratumoral gene expression profiles will be evaluated.

### 2.8 Investigation methods

The tumor will be assessed using contrast-enhanced CT of the abdomen at baseline and each following visit for all included patients. According to the NCI-CTCAE 5.0, any adverse event during the study will be recorded with their types, severity, start and end time, and relatedness to interventions. Blood and tumor tissue specimens before and after PDT will be collected from PDT-treated patients for corresponding tests. After the pre-processing of the blood specimens, PBMC analysis will be performed using flow cytometry to detect the following cells: total T lymphocytes (CD3^+^), cytotoxicity T cells (CD8^+^), CD4^+^ T cells (CD4^+^), and T regulatory cells (Tregs, CD4^+^ CD25^+^ FOXP3^+^). Specimens of tumor tissues will be assessed with an RNA-seq test to investigate the spatiotemporal changes in the expression profile. All patients will receive tumor histological examination [HE stain and immunohistochemistry (IHC)], routine laboratory studies, ECG, chest X scan, ECOG scoring, physical examination, and records of vital signs and concomitant medications. The work timeline and corresponding investigated items are listed in Table 1.

**TABLE 1:** Schedule for the important events in this trial.

Items	Screening phase (within 1 week)	Treatment phase				Follow up phase
		Test group			Control group	
		Cycle 1 (before PDT)	Cycle 1 (after PDT and before ICI plus chemotherapy)	Cycle 1 and beyond (day 1 for ICI plus chemotherapy)	Cycle 1 and beyond (day 1 for ICI plus chemotherapy)	
Informed consent	X					
Inclusion and exclusion criteria	X					
Tumor histological examination	X					
Medical history	X					
Physical examination	X			X	X	X
Vital signs	X			X	X	X
Performance status (ECOG)	X			X	X	X
Concomitant medications	X			X	X	X
Electrocardiogram (ECG)	X					
Chest X scan	X					
Laboratory tests (including blood routine examination, chemistry test, coagulation function, tumor marker, and thyroid function)	X			X	X	X
Pregnancy test (women only)	X			X	X	X
Photosensitizer injection and skin test		X				
Collection and analysis of blood and tumor tissue specimens		X	X			
Tumor assessment by CT	X		X	X (in the cycle 2, 4, 6, and 8)	X (in the cycle 2, 4, 6, and 8)	X
Adverse events assessment		Continuously				X

### 2.9 Follow up

Assessment of treatment response will be performed every two cycles for a maximum of eight cycles. The first tumor assessment will be done within 1 weeks before ICI plus chemotherapy initiation and after PDT if PDT is administered in this subject. Reports of adverse events will be obtained during the whole study period through patient interviews or phone contacts. For PDT-treated patients, blood and tumor specimens will be obtained before light illumination on the day of the PDT operation and 48 h after the PDT operation. Diseases examinations and laboratory studies will be repeated at each cycle of treatment and 1 month after the last dose is received.

### 2.10 Allocation and blinding

Patients will be randomly allocated into the test group and control group at a ratio of 1:1. An independent researcher who will not take roles in patient recruitment will generate a random sequence table using the R software, and the paper strips with allocation results will be kept in a non-transparent envelope. Once the patient is recruited, the doctor will assign the interventions according to his allocated group. As it is difficult and almost impossible to set blind for PDT treatment, this trial is set as open-label.

### 2.11 Sample size estimation

We calculate the sample size based on the ORR. According to the published report, 48.6% of patients receiving ICI plus chemotherapy had an objective response (Shitara et al., 2020). Thus, we expected the ORR to be 50% in our control group. For the patients receiving PDT in combination with ICI plus chemotherapy, we assume that 85% of patients will obtain an objective response based on the experience from our center. Based on these expected proportions, we use a two-sided type I error of 5% with a power of 80% to determine the required lowest sample size. It is supposed that approximately 20% of patients may drop out of the trial. Considering all of these, a total of 60 patients (30 per group) are planned to enroll.

### 2.12 Data collection and management

A data collection group will be established before the initiation of the trial. At least three persons (two data entry clerks and one data administrator) will be employed during the data collection and management. The authorized personnel will enter data using an electronic case report form (CRF) in an EDC-based database (empoweredc, https://www.yunedc.com/). The accuracy of the data entered will be monitored through the quality control tool of the database, and any doubtful data will be rechecked by a third person. The recorded information will be preserved under the database and will be only accessible to the data administrator and other researchers authorized by the principal investigator. Data will not be exported and analyzed until the end of the trial.

### 2.13 Data analysis

All statistical analyses will be performed using the R software (version 4.0.2). The intention-to-treat and per-protocol approaches will be used for the analysis of the primary outcomes. The as-treated analysis will be used for safety analysis. Continuous data will be presented as mean with standard deviation or median with interquartile range. Categorical data will be presented as numbers with percentages. Student’s t-test or Mann-Whitney *U*-test will be used for the comparison of continuous data, while chi-squared test or Fisher’s test will be used for categorical data. For patients in the test group, the paired *t*-test or Wilcoxon signed-rank test will be used to analyze the difference in PBMC data before and after PDT treatment. The analysis of the gene expression profile will follow the standard procedure for transcriptomic analysis, and a protocol will be developed once the specimens are collected. The survival plot will be drawn for PFS using the Kaplan-Meier method. Log-rank test will be used to compare the difference between the two groups, and Cox regression analysis will be performed to estimate the hazard ratio with 95% CI. All statistical tests will be two-sided, and a significant level will be set at 5%.

### 2.14 Data monitoring

The trial process will be monitored by the institutional data safety monitoring committee at regular intervals (at least annually). All event reports of toxicities, treatment discontinuation, and transformation will be recorded and submitted to the data monitoring committee. The primary investigator will decide to continue or abort the trial based on the safety assessment from the data safety monitoring and ethics committee.

### 2.15 Confidentiality

All participants will be allocated a unique code for identification once they are included. All specimens and forms will be securely maintained, and electronic data will be stored in the database with an encryption technique. Only authorized personnel can access the data. The personal information of participants will be confidential and never shared with any third-party organizations. The primary investigator is the person who is finally responsible for the security of the data.

## 3 Discussion

Although a lot of previous studies have demonstrated an enhanced immune response by PDT, almost all of them are preclinical evidence from animal models (Krosl et al., 1995; Cecic et al., 2006; Wang et al., 2013; Li et al., 2019). Further high-quality evidence from randomized control trials is needed to clarify the immune-stimulated role of PDT in a clinical condition. Here we perform a randomized control trial grouped by PDT in combination with ICI plus chemotherapy versus ICI plus chemotherapy, aiming to evaluate the synergistic effect of PDT with immunochemotherapy. Successful implementation of this trial will help to find an immunologic adjunct for ICIs-based therapy. At present, the scheme of ICI plus chemotherapy is used as a first-line treatment for patients with metastatic gastric cancer, but the efficacy of this combination is still limited, especially in patients with unclear PD-L1 status. In the KEYNOTE-062 study, pembrolizumab plus chemotherapy was shown no superiority to the chemotherapy alone in gastric cancer patients with a PD-L1≥1 (Shitara et al., 2020). Another study (ATTRACTION-4) evaluated the combination of nivolumab plus chemotherapy in an Asian population. PD-L1 expression is not used as the criterion for the selection of patients in this study, and better results were found in the ORR and PFS but not the OS when comparing ICI plus chemotherapy with chemotherapy alone (Boku et al., 2020). Based on these facts, it is meaningful to improve the therapeutic effects of ICI by appending an adjunct. As a well-tolerated treatment in most cancer patients, a combined strategy with PDT will not significantly increase the risks of safety in theory. This is our foundation for conducting this trial.

Our cancer center has rich experience in PDT treatment in digestive tract tumors, which will provide support to assure the subjects receiving a homogeneous treatment in this trial. We used ICI plus chemotherapy as the standard scheme, which is in accord with the beneficence principle in medical ethics. In addition, this gives a promise of enough interested patients for recruitment. In this trial, we will only include patients with age ≤65, and poor health conditions will be excluded. This is based on the consideration that elderly patients with poor conditions are easier to drop out due to their intolerance of the assigned treatments. Decreasing the ratio of drop-outs can avoid the negative influence caused by it on data analysis and increase the validity of our results.

There are also some limitations in this trial. Firstly, chemotherapy will be concurrently applied with ICI in the schemes of this trial. This means this trial can just identify the effects of PDT for ICI combined with chemotherapy. How these effects come and whether PDT can synergize with either of the ICI and chemotherapy or both of them will be hard to explain. In the future, we will conduct further studies to clarify these when the roles of PDT in the tumor microenvironment are more demonstrated. Secondly, this is an open-label study, and blinding will not be used during the study. The risk of bias caused by this will inevitably impact the results to some extent. Finally, the sample size is small. It may be difficult to perform subgroup analysis with these subjects.

This trial is the first study to explore the clinical significance of PDT in improving the outcomes of ICI plus chemotherapy in patients with advanced or metastatic gastric cancer. This paper provided an interpretation of its design and methodology. The results of this trial will help us know the roles of PDT in gastric cancer and may give a novel combined strategy for immunotherapy.
